# Detection and measurement of butterfly eyespot and spot patterns using convolutional neural networks

**DOI:** 10.1371/journal.pone.0280998

**Published:** 2023-02-13

**Authors:** Carolina Cunha, Hemaxi Narotamo, Antónia Monteiro, Margarida Silveira

**Affiliations:** 1 Institute for Systems and Robotics (ISR), Instituto Superior Técnico (IST), University of Lisbon, Lisbon, Portugal; 2 Biological Sciences, National University of Singapore, Singapore, Singapore; Universita degli Studi di Perugia, ITALY

## Abstract

Butterflies are increasingly becoming model insects where basic questions surrounding the diversity of their color patterns are being investigated. Some of these color patterns consist of simple spots and eyespots. To accelerate the pace of research surrounding these discrete and circular pattern elements we trained distinct convolutional neural networks (CNNs) for detection and measurement of butterfly spots and eyespots on digital images of butterfly wings. We compared the automatically detected and segmented spot/eyespot areas with those manually annotated. These methods were able to identify and distinguish marginal eyespots from spots, as well as distinguish these patterns from less symmetrical patches of color. In addition, the measurements of an eyespot’s central area and surrounding rings were comparable with the manual measurements. These CNNs offer improvements of eyespot/spot detection and measurements relative to previous methods because it is not necessary to mathematically define the feature of interest. All that is needed is to point out the images that have those features to train the CNN.

## Introduction

Eyespots are salient color pattern stimuli with multiple rings of contrasting colors that mimic vertebrate eyes. They are used by a variety of animals primarily to intimidate or startle predators or to deflect predator attacks to dispensable areas of the body [1]. Eyespots have been studied primarily in the lepidoptera, where different modes of defense are found in different species, and where eyespots are also used in sexual signaling [2–4]. Eyespots in nymphalid butterflies have a single origin [5], and they may have evolved from simpler pattern elements, spots [6]. Spots are simple patches of color contrasting against the background color of the wing. It is unclear how many times spots have evolved independently in butterflies and what exact ecological function they serve.

The accurate measurement of spots and eyespots has become a routine task for researchers who study the ecological role of these traits in butterflies. For example, different sizes of eyespots found in males and females, hinted at their role in sexual signaling [7], whereas different sizes of eyespots in dry and wet seasons forms of the same species of butterfly hinted at the presence of different predator guilds in each season shaping eyespot size [8].

Measuring spots and eyespots has also become important for researchers who explore mechanistic questions about their development. For instance, in order to probe the mechanism of sexual size dimorphism or seasonal phenotypic plasticity, researchers use a variety of hormone and drug injections during development to test how they affect the final size of the eyespots [9, 10]. Eyespot and spot measurements are also performed to test the role of candidate genes in the development of these traits using genetic perturbations [11–13]. Having the ability to perform quick measurements on spots/eyespots is, thus, useful to accelerate the pace of research around these traits.

Previous approaches for automatic eyespot detection and measurement [14] relied on a sliding window approach where carefully selected features exploiting symmetry and circularity were measured. These features were then fed to an SVM classifier for detection, after which the different circular rings were measured with a 1D Hough Transform for circle detection.

Convolutional neural networks (CNN) [15] are currently the most widely used deep learning algorithms for image analysis, having outperformed traditional algorithms in many image analysis problems like image classification, object detection or segmentation [16, 17]. The main advantage of CNNs over previous methods is their ability to automatically extract from the images the most relevant features of the patterns of our choice, requiring no manual feature extraction.

While CNNs have been used in the past for purposes of butterfly species identification [18–22], they have not been used to identify specific wing patterns, such as spots and eyespots, regardless of species identity.

CNNs have been used for the analysis of other circular patterns such as nuclei in digital pathology images. A popular approach is to segment the images into two classes (nucleus and background) with the U-net, the most widely used CNN for semantic image segmentation. Since semantic segmentation cannot separate touching or overlapping nuclei, post-processing approaches such as watershed [23] or H-minima transform [24] are commonly used. Another possibility to separate the segmented touching nuclei is to use a three-class U-net to segment not only the nuclei from the background, but also the boundary at each nucleus. For example [25] used a three-class U-net and proposed a new loss function that considers both class imbalance and cell shape. Fully Convolutional Networks (FCN), a type of CNN composed only of convolutional layers and without fully connected layers, have also been used. For instance [26] used a FCN to predict the nuclei segmentation maps followed by watershed postprocessing while [27] used a FCN to predict a distance map to centroids and boundaries of nuclei. Instance segmentation methods such as the Mask R-CNN jointly detect and segment thus solving the splitting problem. Mask R-CNN was used in [28, 29] however their training is slow and the segmentation masks are not as accurate as those of U-net. In this type of image segmentation, however, the images of the cell nuclei, and cell background, are similar, which is not the case with the images of our diverse species of butterflies.

In this work, we use CNNs for both spot/eyespot detection and measurements. We first use a dataset of images of different species of butterflies with a variety of spots and eyespots for training and testing three state-of-the-art object detection CNN methods. Then we test the best CNN to detect eyespots in a different image dataset consisting of images of many individuals of a single species. In this second image dataset we also train and test a new CNN to segment and measure different eyespot areas.

## Materials and methods

### Data

**Fig 1:**
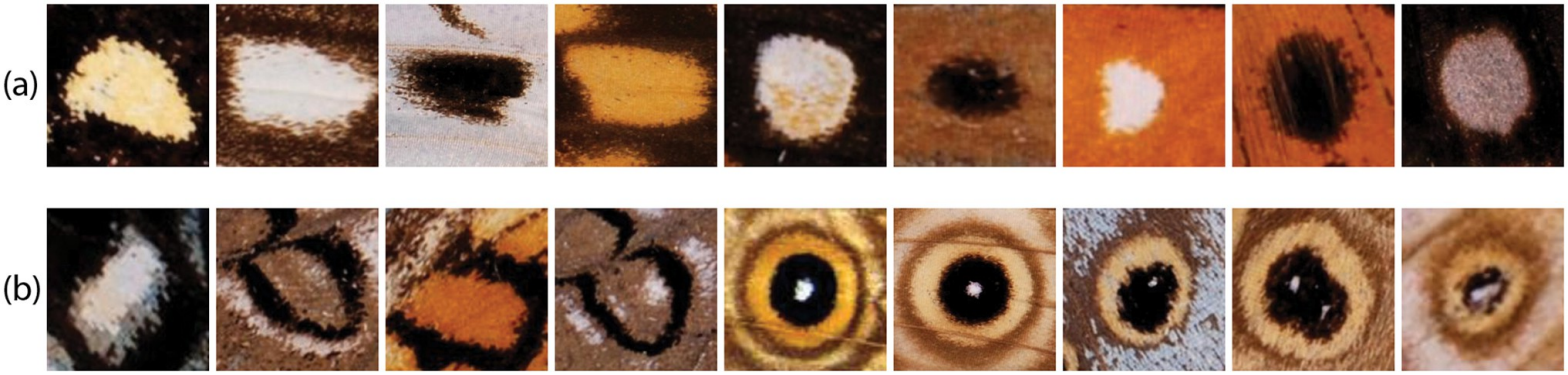
Examples of the types of pattern element present in dataset 1. Spots are pattern elements that develop a single spot of color. Eyespots are pattern elements that develop spots and rings of color. These include discal and marginal eyespots. The first are eyespots that develop around a cross-vein and are found in the center of the wing, and the second develop closer to the margin of the wing. (**a**) Spots. (**b**) Eyespots.

Two different datasets were used in our experiments. For the spot and eyespot detection task we used a dataset (dataset1) with 4707 butterfly images of different species, 690 of which were images of butterflies without any spots or eyespots, and the remaining 4017 contained one or more spots or eyespots. In total, these images contain 17382 spots and 16571 eyespots. For each butterfly image containing spots/eyespots, the right-wing pattern elements had been identified manually in two previous studies [5, 6] and their center position and size recorded. The type of pattern element was also identified. Fig 1 illustrates the variability of the different types of pattern elements.

For the eyespot measurement task we used a different dataset (dataset 2) containing 64 images of the ventral forewing of a single species, *Bicyclus anynana*. In each image, two marginal eyespots, a small and a bigger one, had been identified manually and their center coordinates recorded. Additionally, for each eyespot, the total area, up to the outer perimeter of the orange ring, and the white center area had been measured in *mm*^2^.

**Fig 2:**
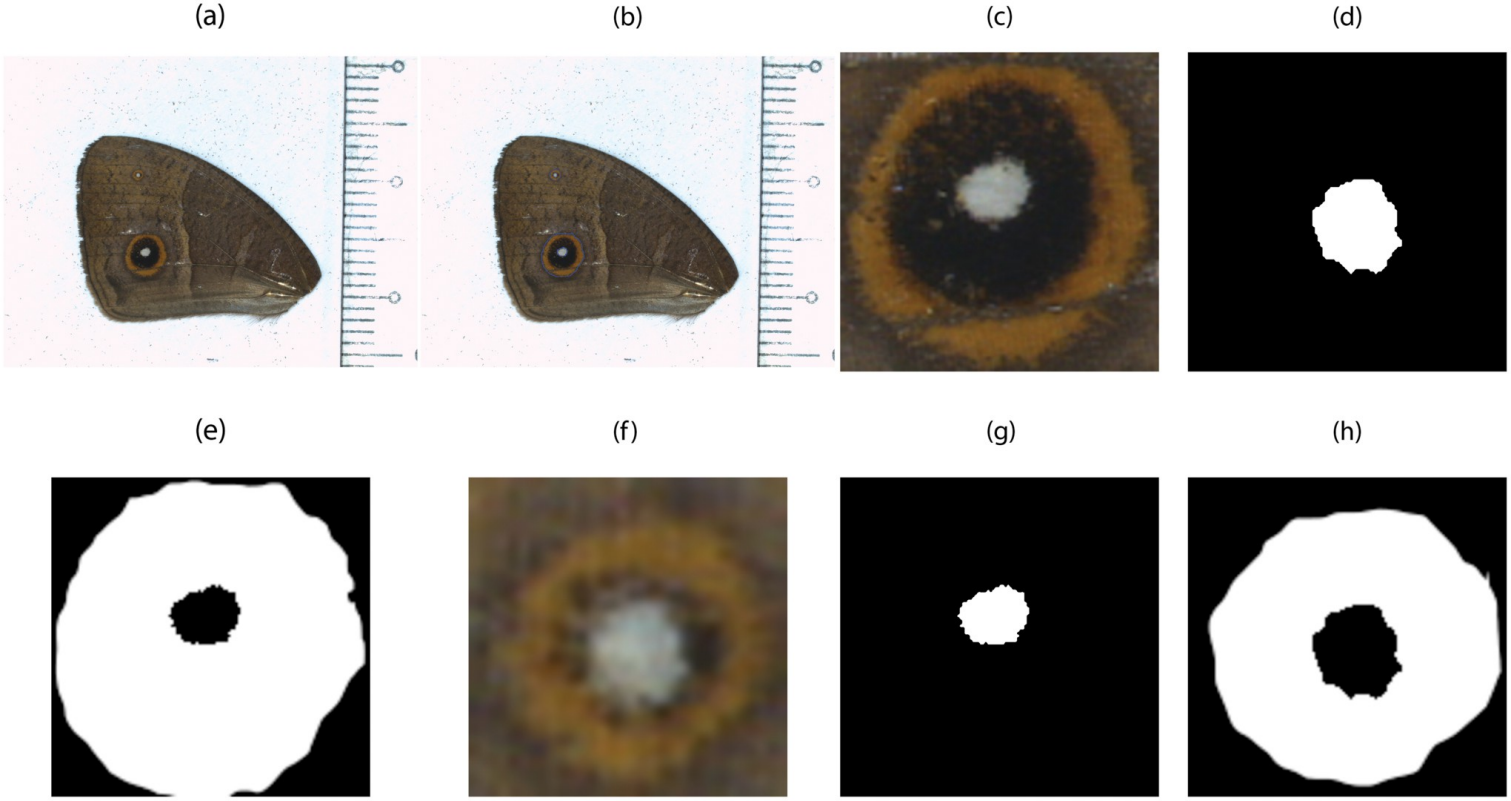
Illustration of ground truth created for *Bicyclus anynana* wing image, where white regions correspond to areas we wish to measure and for which we also have obtained manual measurements. (**a**) The original RGB image with two marginal eyespots. (**b**) Image with ground truth circles superimposed on each eyespot. (**c**) Large eyespot crop resized to 128x128 pixels. (**d**) Large eyespot center ground truth mask. (**e**) Large eyespot rings ground truth mask. (**f**) Small eyespot crop resized to 128x128 pixels. (**g**) Small eyespot center ground truth mask. (**h**) Small eyespot rings ground truth mask.

From the visual inspection of each crop, ground truth segmentation masks were manually created with white regions corresponding to the eyespot color rings (black and orange rings combined) and to the center. Fig 2 shows some examples.

### Image analysis

The analysis comprised two steps that use CNN’s: (1) spot/eyespot detection and (2) spot/eyespot measurements. First, the butterfly image is fed to the detection CNN, which provides as output the bounding boxes of the detected spots/eyespots and corresponding types. Then, for each pattern element that was detected in step (1) the corresponding cropped image will be extracted from the input image and fed to the second CNN. Since CNN input images must all have the same size and the spots/eyespots have a wide range of sizes, the patches are resized to a common size before being fed to the measurements CNN. The CNN then performs the segmentation of the pattern element and outputs binary segmentation masks corresponding to each of the pattern elements we wish to measure.

**Fig 3:**
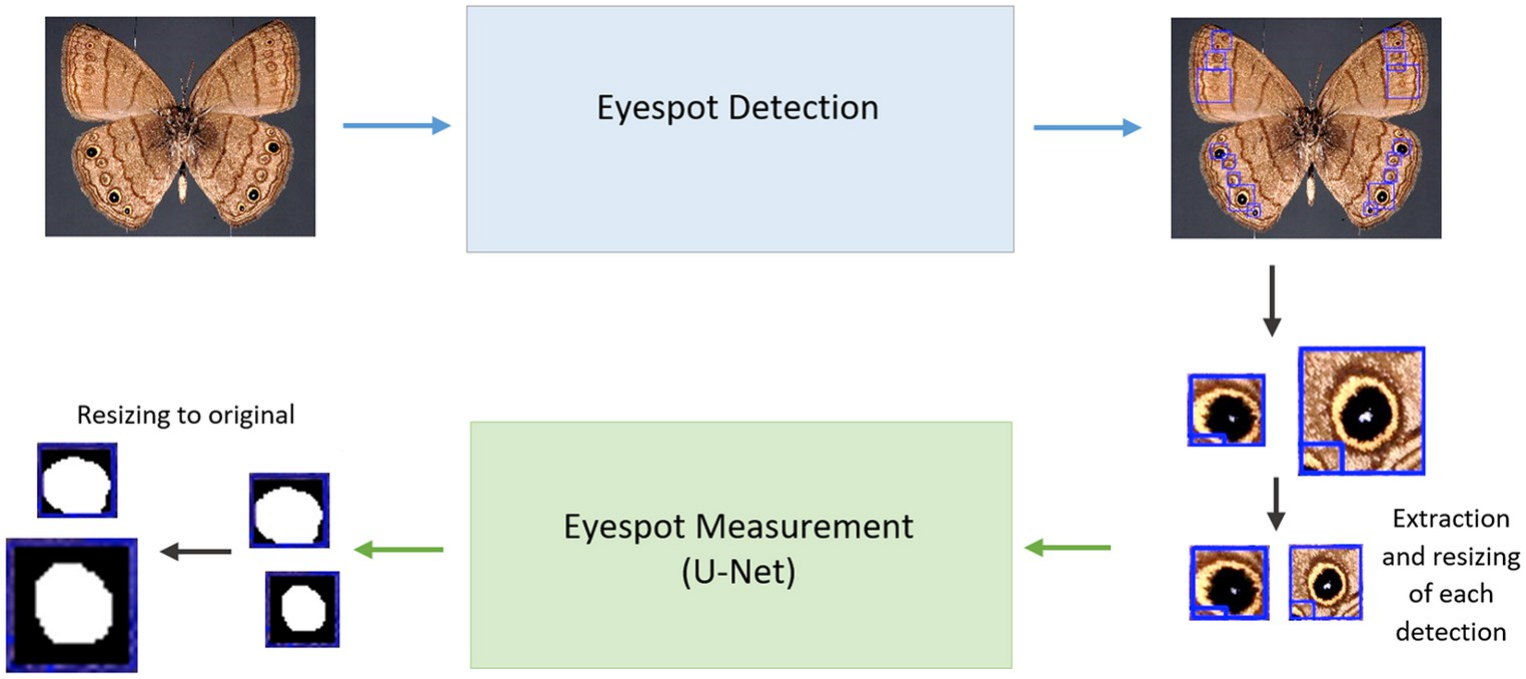
Overview of the two CNNs approach for first detecting and then measuring, spots or eyespots on images of butterfly wings.

In Fig 3, a basic scheme with the overview of the entire detection + measuring system is illustrated for an eyespot-bearing butterfly.

#### Spot and eyespot detection

For spot and eyespot detection we compared the performance of three state-of-the-art deep learning algorithms for object detection and classification: YOLO [30], RetinaNet [31] and EfficientDet [32]. Each of these three models detects objects in the images and provides, for each detected object, a bounding box and class probabilities. YOLO, RetinaNet and EfficientDet are fast, compared to other object detection methods like RCNN, because they use the same CNN to predict the bounding boxes and the class probabilities for those boxes.

YOLO first divides the image into a grid and then predicts bounding boxes and class probabilities for each cell in the grid. Both tasks are solved as a regression problem, i.e, YOLO finds a mapping between the input image pixels and the coordinates and sizes of the bounding boxes and the respective class probabilities. We used YOLO v3, which uses the Darknet as a feature extraction CNN. This is a CNN with 53 convolutional layers and 3 prediction heads, each processing the image at a different spatial scale. For the detection task 53 more convolutional layers are added, making YOLO a fully convolutional network (FCN) with a total of 106 layers.

RetinaNet is a one-stage detector that performs feature extraction using a ResNet [33] and a Feature Pyramid Network (FPN). We used a ResNet-50 which contains 50 layers. The FPN is built on the top of the ResNet and contains 5 levels. Additionally, RetinaNet contains two sub-networks, one for bounding box prediction and another for classification. These sub-networks are small fully convolutional networks (FCNs), each contains only 5 layers, and they share parameters between them. They perform bounding box regression and classification on top of each layer of the FPN which allows to detect objects at several scales. Moreover, RetinaNet uses the Focal loss to deal with the problem of class imbalance. Focal loss decreases the contribution of easy samples to focus on foreground samples that are typically underrepresented.

EfficientDet is a single-stage object detection model that uses the EfficientNet [34] as backbone network. Moreover, it includes a bi-directional FPN (BiFPN) containing learnable parameters which allows to combine input features at various scales. Based on the output layers of EfficientNet, the BiFPN layers perform feature extraction and fusion. Thereafter, the BiFPN layers’ output is fed to a network that predicts the bounding boxes and objects classes. EfficientDet includes several models with different complexities (D0, D1, D2, D3, D4, D5, D6 and D7). With increasing complexity the detection performance is also increased. The layers of the backbone network, of the BiFPN modules and of classification and bounding box prediction networks, all depend on the complexity of the model. For instance, for the EfficientDet-D0 the backbone network contains 237 layers while the BiFPN and classification and bounding box prediction networks contain 3 layers each.

After all the bounding boxes have been predicted, YOLO, RetinaNet and EfficientDet perform non-maximum suppression (NMS) to remove duplicate detections. In this step, boxes with high confidence scores are selected, and the ones that overlap are removed, i.e. the ones that have IoU with the selected boxes above a predefined threshold.

Three alternatives were investigated for each detection model: training the model with two classes, corresponding to the two types of pattern element (spots and eyespots), and training the model with only one class, either detecting all pattern elements without differentiating the type, or detecting only marginal eyespots, which are the most frequent, and the only ones present in dataset2.

#### Eyespot measurements

We treated the eyespot measurements problem as an image segmentation problem, and used a CNN to segment the different eyespot areas we wished to measure. Unlike previous approaches that only detect circular eyespots [14], this approach makes no assumptions on the shape of the eyespots.

For segmentation we used the U-Net [35], a widely used CNN designed for image segmentation. Although this encoder-decoder network was proposed, and is used primarily, for biomedical images, it can be used with images of any kind. Our U-Net was trained using patches containing the detected eyespots and not the whole image. The eyespot patches were resized, since U-Net inputs must be of the same size. For each input patch, this network will output an image, of the same size, with binary segmentation masks for the different areas of the eyespot, namely the black and orange rings together, and the white center. Once an eyespot has been segmented, areas of the different segmentation masks are obtained by adding the pixels contained within the binary segmentation mask that is obtained at the output of the U-Net. It is also necessary to perform an inverse resize operation to the original dimensions that the crop of each eyespot had in its wing image. Once the area of the center and that of the surrounding color rings are determined, the total eyespot area is obtained by adding the two quantities.

We implemented two versions of the U-Net that can provide us with the same set of measurements, namely, area of the white center, and of the surrounding color rings. One segments only the two color rings from the rest of the image (two-class) and the other segments center, color rings, and background (three-class). Both versions are trained using the categorical cross entropy (CCE) as the cost function. We used the unweighted version of this cost function, which is more commonly used, and also a weighted version, termed Weighted Cross entropy (WCCE), which includes class weights. Using class weights is important to deal with the fact that there are large differences in size of the areas to segment, for example the center class has much fewer pixels, compared to the color rings and background classes. Using class weights in the loss function allows us to increase the weight of the minority classes to compensate for these differences.

The code for the YOLO, RetinaNet, EfficientDet, and U-Net CNNs is available for download at https://github.com/Margarida-Silveira/Butterfly_CNN.

#### Evaluation

CNNs models for the detection of the different pattern elements are evaluated using Average Precision (AP) and mean Average Precision (mAP). AP is the most commonly used metric to evaluate the performance of object detection algorithms. It is computed as the area under the Precision-Recall curve for Intersection over Union (IoU) thresholds between 0.5 and 0.95. mAP is the mean of AP for all the object classes, in this case the type of pattern elements (spots or eyespots).

Precision is the proportion of correctly classified pattern elements of a given type out of all the pattern elements identified by the model as that type, computed as:

$$\begin{array}{c} Precision=\frac{TP}{TP+FP} \end{array}$$ (1)

and Recall is the proportion of correctly classified pattern elements of a given type among all the pattern elements from that type, and is computed as:

$$\begin{array}{c} Recall=\frac{TP}{TP+FN} \end{array}$$ (2)

In the previous equation TP (true positives) is the number of correctly classified pattern elements from a given type and TN (true negatives) is the number of correctly classified samples from all the other types. Similarly, FP (false positives) is the number of incorrect classifications of a given pattern element type and FN (false negatives) corresponds to the total number of missed detections from all types of pattern elements.

A pattern element is considered a TP if the Intersection over Union (IoU) between its bounding box and any ground truth bounding box (manually identified) of the same type of pattern element is above or equal a given threshold, with IOU computed as the area of intersection divided by the area of union:

$$\begin{array}{c} IoU=\frac{\text{Area}\;\text{of}\;\text{Intersection}}{\text{Area}\;\text{of}\;\text{Union}} \end{array}$$ (3)

In case multiple detections of the same object occur, the one with highest probability is counted as positive while the rest are counted as negatives.

U-Net segmentation results were evaluated with Accuracy, macro F1-score and IoU. Accuracy measures the percentage of pixels that are classified correctly and the F1-score is the harmonic mean between pixel wise Precision and Recall, computed as:

$$\begin{array}{c} F1=2\cdot \frac{Precision\cdot Recall}{Precision+Recall} \end{array}$$ (4)

The macro F1 is the average (unweighted) of the F1 score obtained for each class. IoU is computed in the same way as for the CNNs detections, but in this case, it is computed between ground truth segmentation and predicted segmentation, and not bounding boxes. Finally, area measurements were obtained from the segmented images, converted to *mm*^2^ and compared to manual measurements.

## Results

### Eyespot detection

Dataset1 was randomly divided into 90% (3615 images) for training and 10% (402 images) for testing. For each image its pixel RGB values were divided by 255 to guarantee values in the 0 to 1 range. The results presented below were obtained by applying the trained CNN models on the test data. This test set includes 402 images of whole butterflies with several spots/eyespots. In these butterfly images, there were 33953 pattern elements of the 2 types divided as shown in Table 1. Furthermore, 88% of all eyespots (14553 vs 2018) were marginal eyespots.

**Table 1:** Number of pattern elements of each type in the training/validation and test set for dataset1.

	Training Set	Test Set
**Spot**	15644	1738
**Eyespot**	14914	1657
**All**	30558	3395

**Fig 4:**
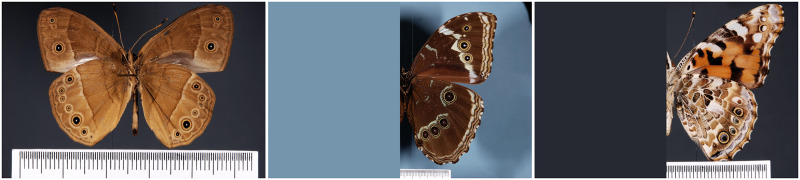
Examples of images from dataset1. Image of a complete butterfly and two butterfly images with left wing erased.

Since only the right wing pattern elements were previously annotated, we manually annotated the elements in the left wing for the 402 test images using labellImg software [36]. To avoid the burden of also annotating the elements in the left wings for the training images, we instead erased the left part of each training image, as illustrated in Fig 4.

The YOLO, RetinaNet and EfficientDet networks used are based on publicly available implementations released in the GitHub repositories [37–39] respectively.

Each network was trained with the Adam optimizer using an initial learning rate of 1e-04, *β*_1_ = 0.9 and *β*_2_ = 0.9999, and a batch size of 8. Training was stopped when the number of iterations reached 100 or earlier, if the loss function did not decrease for the validation set for more than 10 epochs (the patience hyperparameter). For all models, duplicate detections were removed using Non Maximum Suppression (NMS). There are many spots or eyespots that overlap, however in most cases the overlap is small. Accordingly the NMS threshold was set to 0.3.

We trained the models using online data augmentation to increase the diversity of our training data. Our data augmentation strategy included random distortions of the hsv space and also horizontal flipping to make sure the network was trained with left-wing eyespots also and not affected by the removal, from the training images, of the wings that were not annotated.

The average precision (AP) and the mean average precision (mAP) values achieved in the test data are presented in Table 2.

**Table 2:** Scores achieved by YOLO, RetinaNet and EfficienDet-D0 models for the three eyespot detection tasks on the test data from dataset1.

Method		AP	mAP
YOLO	Spot	0.4799	0.6562
	Eyespot	0.8326	
	All	0.8330	0.8330
	Marginal eyespot only	0.8472	0.8472
RetinaNet	Spot	0.4933	**0.6824**
	Eyespot	**0.8716**	
	All	**0.8598**	**0.8598**
	Marginal eyespot only	**0.8689**	**0.8689**
EfficientDet-D0	Spot	0.2687	0.5087
	Eyespot	0.7487	
	All	0.7561	0.7561
	Marginal eyespot only	0.7528	0.7528

We also tested the RetinaNet models trained with data from dataset1 on images from dataset2. The results are presented in Table 3.

**Table 3:** Scores achieved by the three RetinaNet models for eyespot detection on dataset2.

	AP	mAP
**Spot**	0	
**Eyespot**	0.8437	-
**All**	**0.8471**	**0.8471**
**Marginal eyespot only**	0.8269	0.8269

### Eyespot measurements

To train the U-Net to perform automatic eyespot measurements we randomly divided dataset2 into 80% images for training (101 eyespots) and 20% images for testing (24 eyespots). From the center X,Y coordinates and total area annotations in dataset2 we were able to create square eyespot cropped images, which were resized to 128x128 pixels.

The results presented below were obtained by applying the U-Net trained models on the test set. The class weights used in WCCE loss function for two-class data were 1.75 and 1, corresponding to the eyespot rings area and to the rest of the image area, respectively, and for the three-class data they were 2, 25 and 1, for the eyespot rings, center, and background area classes, respectively. For the two-class model, to isolate the center region, we computed the negative of the segmentation mask and then eliminated the components connected to the image boundary using mathematical morphology.

Our U-Net was trained with the Adam optimizer with an initial learning rate of 1e-04, *β*_1_ = 0.9 and *β*_2_ = 0.9999, and a batch size of 8. The network was trained for a maximum of 100 epochs, using an early stop criterion monitoring the validation loss with a patience of 10.

**Fig 5:**
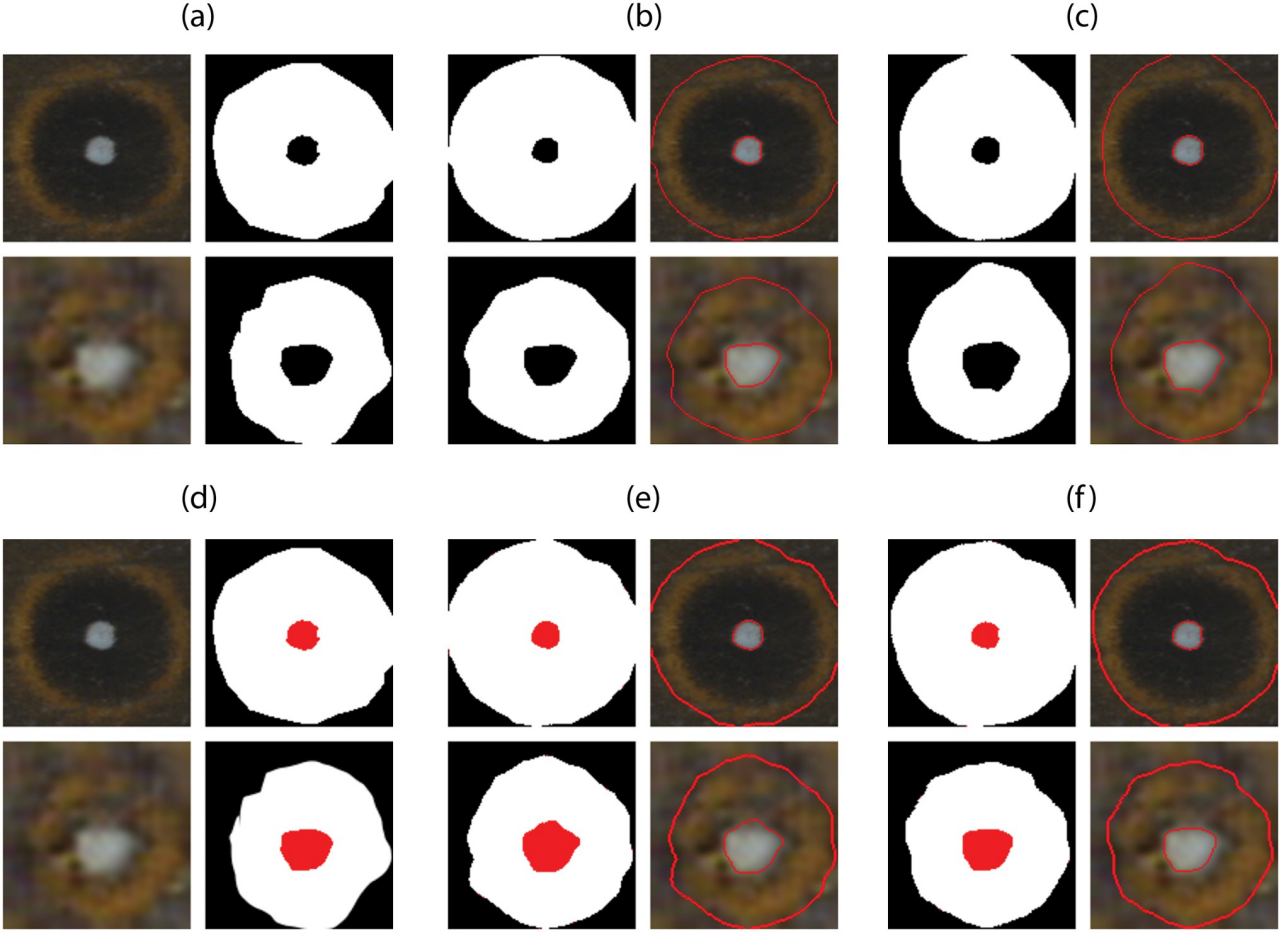
Example of U-Net segmentation results using different number of classes and cost functions. (a) Original RGB eyespot image and its two-class ground truth segmentation, (b) Predicted segmentation using CE loss function and corresponding contours. (c) Predicted segmentation using weighted CE loss function and corresponding contours. (d) Original RGB eyespot image and its ground truth segmentation, (e) Predicted segmentation using CE loss function and corresponding contours. (f) Predicted segmentation using weighted CE loss function and corresponding contours.

Table 4 presents the accuracy, F1 and IoU results of U-Net segmentation experiments with two-class data and with three-class data. In Fig 5 we illustrate some of these results.

**Table 4:** Evaluation of U-Net segmentation models trained with different cost functions (CCE and WCCE).

	Acc	F1	IoU
Two-class CCE	0.925	0.904	0.829
Two-class WCCE	0.934	**0.924**	**0.862**
Three-class CCE	0.935	0.910	0.845
Three-class WCCE	**0.942**	0.915	0.855

Acc = accuracy; F1 = macro F1-score; IoU = Intersection over union.

For comparison with our method, we also trained a U-net to segment the eyespots on the entire butterfly wing images. The ground truth was created from the manual GT for the eyespots in dataset2, which were placed at the corresponding eyespots position on the wing. To train this U-net we resized the images and corresponding GT masks to 640x640. For larger image sizes, training of the U-net model did not converge and for smaller sizes the eyespot areas were too small to segment. After segmentation, we used connected components labelling to identify the different eyespots. There was no need for further post-processing to separate the eyespots, watershed for example, since there is no overlap between them. Training used the same weighted loss function and optimizer as our U-net. The class weights for eyespot rings and center used in the WCCE loss function were increased since a much larger percentage of the image pixels are now background. For the two-class data the weights were 50 for the eyespot rings and 1 for the rest of the image area, and for the three-class data they were 600, 25, and 1, corresponding to the eyespot rings, center, and background, respectively. Table 5 presents the accuracy, F1 and IoU results of both U-Net segmentation models with two-class data and with three-class data.

**Table 5:** Comparison of our U-Net segmentation models with U-net models trained on the entire wings.

		Acc	F1	IoU
**Proposed**	Two-class WCCE	0.934	**0.924**	**0.862**
**U-net**	Three-class WCCE	0.942	0.915	0.855
**Whole wing**	Two-class WCCE	**0.998**	0.422	0.329
**U-net**	Three-class WCCE	0.993	0.650	0.333

Acc = accuracy; F1 = macro F1-score; IoU = Intersection over union.

For the best segmentation models, using two classes and three classes, we measured the areas of the eyespot rings and center in pixels and converted those measurements to squared millimeters (1 *mm* = 28.944 px). Then we computed the errors between manual and automatic area measures. These results are presented in Table 6.

**Table 6:** Average error and error standard deviation between manual and automatic measurements of the total eyespot and center areas. Relative errors and errors in *mm*^2^ are presented.

	U-Net two-class WCE		U-Net three-class WCE	
	Center	Total	Center	Total
**Relative Avg. Error [%]**	29.10	6.65	27.37	**5.23**
**Relative Error std [%]**	14,78	**4.10**	19.14	4.64
**Avg. Error [*mm*^2^]**	0.05	0.28	0.04	0.16
**Error std [*mm*^2^]**	0.03	0.24	0.03	0.15

## Discussion

**Fig 6:**
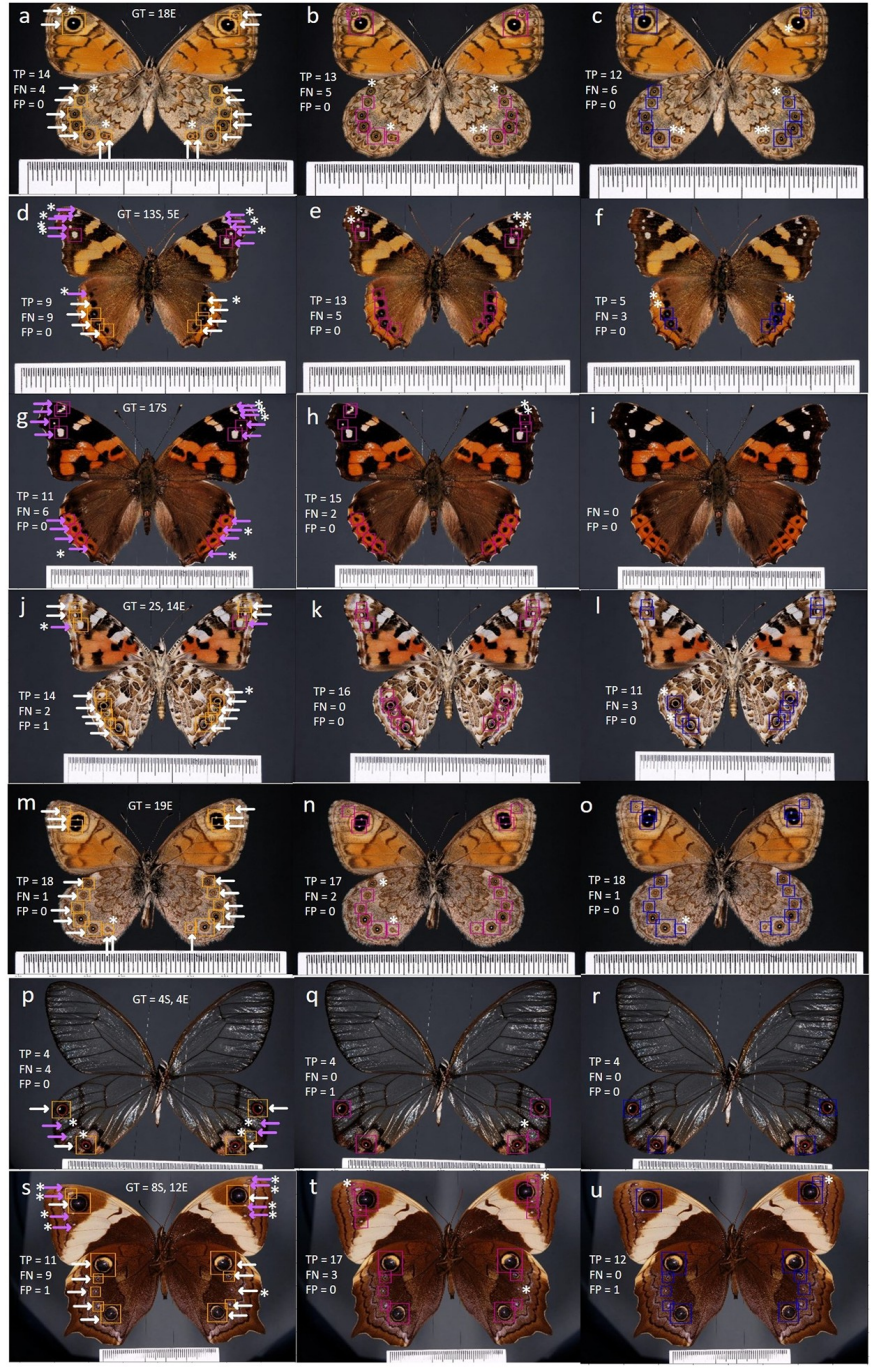
RetinaNet detection examples. Left column shows detections of the two-class model (spots in pink and eyespots in orange), middle column shows detections of one-class (no distinction between spot or eyespot types) and right column shows detections of “Marginal eyespots only” RetinaNet. The first image in a row is annotated for the ground truth (GT) in terms of total number of spots (S) and eyespots (E), with eyespots marked with white arrows, and spots with pink arrows. In the other images we score the true positives (TP), the false negatives (FN) and the false positives (FP) and we add an asterisk (*) next to the undetected or misclassified pattern elements.

The detection models were able to correctly detect a large number of spots and eyespots, despite the variability in the shape and color of these patterns and in the number of pattern elements per image. Spots/eyespots were detected even in cases where there is little contrast between these patterns and the background, and in cases where spots/eyespots were overlapping. As illustrated in Fig 6, most bounding boxes were very close to the ground truth ones, perfectly encapsulating the pattern elements. The majority of missed detections occurred for very small or overlapping spots/eyespots. There were also a few detections that were counted as false positives but that corresponded to eyespots that were not included in the manually annotated ground truth.

From the analysis of Table 2 we can conclude that the two-class models obtained better detection results, in terms of AP, for eyespots than for spots, probably because spots have higher pattern variability. With all data belonging to the same class, the networks only have the task of detecting where the pattern elements are, without having to find out which class they belong to. Thus, the mAP obtained with one class YOLO, RetinaNet and EfficientDet was, respectively, 0.83, 0.86 and 0.76 which is higher than the mAP obtained with the corresponding two class models: 0.66, 0.68 and 0.51. The YOLO and RetinaNet models trained to detect marginal eyespots achieved an AP of 0.85 and 0.87, outperforming the other two models tested here (two-class and one class to detect both spots and eyespots).

RetinaNet outperformed YOLO and EfficientDet as the best model to detect both spots and eyespots. Moreover, we concluded that EfficientDet is the worst-performing model. It presents a difference of 10.37% and 11.61% compared to the best performing method for spots/eyespots and marginal eyespots detection, respectively. To perform a fair comparison between the three models, we considered EfficientDet-D0 model, which takes images of size (512,512,3) as input. One possible explanation for the bad results obtained with EfficientDet is that EfficientDet-D0 is not complex enough to detect more spots and eyespots. We think that the performance of EfficientDet can be improved by training more complex models, such as EfficientDet-D7 that outperforms RetinaNet in other detection tasks [32]. However, EfficientDet-D7 requires an input of size (1536,1536,3) which is much higher than the input size of the YOLO and RetinaNet models trained in this work (which have a size of 416,416,3). Finally, both YOLO and RetinaNet perform well in the two one-class tasks to detect spots/eyespots and marginal eyespots and, as expected [31], RetinaNet is better than YOLO.

The results of the RetinaNet models to detect the eyespots in dataset2, shown in Table 3, are not as good as the results of these models for the eyespots in dataset1, which was foreseable, since the training set (dataset1) has no examples from this butterfly species and there is little contrast between the eyespots and the rest of the wing. Furthermore, the eyespots in these images have different dimensions from the ones in the training images. This is a problem for RetinaNet because in RetinaNet the size of the bounding boxes is learned from the data. These differences in size reduce the IOU between predictions and ground truth and also AP, although most eyespots in the test set have been detected. The best model was the 1 class spot/eyespot detection model which was able to achieve an AP of 0.85 on images from dataset2. Although dataset2 only contains eyespots, there are some small eyespots that resemble spots, which are not detected by the “Marginal eyespots only” model. This explains the smaller AP achieved by this model (0.83) compared to the 1 class spots/eyespots detection model.

The U-Net models were able to segment the two areas within an eyespot, white center and black and orange rings combined, even though the training set was small. Both models (two-class and three-class) had similar overall segmentation performance as shown in Table 4, achieving accuracies over 95%, F1-score over 91% and IoU over 82%. For both models, the results improved with the use of weighted categorical entropy.

We compared our U-net with a U-net trained on the entire wing images. The results in Table 5 show that the accuracy of the whole image U-net model is higher than our U-net. However, this increase does not mean that the model is better. This increase occurs because the background area, which comprises the actual background and wing areas not containing spot or eyespots, is much larger than the other areas we wish to segment, and the model is biased toward segmenting everything as background. In fact, if we look at the macro F1-score which is the unweighted average of the F1-score for the three classes, we see that it decreased considerably because the eyespot is badly segmented. The same happened with mean IOU which decreased dramatically for the U-net trained on the entire wing. Even though we used class weights to try and compensate for the imbalance, this was not enough to obtain a good model. In addition, the whole wing U-net model is more complex, it has more parameters as the input images are larger, and there are fewer images to train this model than if we use the individual patterns. Both factors contribute to overfitting and lack of generalization. Furthermore, this U-net is also much slower to train.

The differences between manual area measurements and automatic ones are shown in Table 6. The average relative error for the total eyespot areas is small, 5.2% for the weighted three-class model and a little higher, 6.7%, for the weighted two-class model. These errors increase for the spot center (27.4% and 27.4%) because the spot center is very small thus the impact of a single mislabeled pixel on the errors is high. Automatic area measurements in *mm*^2^ were comparable with the manual measurements.

## Conclusion

In this work we investigated the use of convolutional neural networks to automatically detect and measure eyespots in images of butterflies. Three CNNs were trained to identify and distinguish spots and eyespots across the whole wing (dataset1) or marginal eyespots alone (dataset2), in whole photos of different species of butterfly. Another CNN was trained to segment eyespot patterns into two different areas (center and remaining rings) in photos of a single species of satyrid butterfly. Spots and eyespots were, for the most part, accurately identified and segmented by the CNNs, and the automatic measurements were comparable with the manual measurements. These CNNs, once implemented together with a graphical user interface, where imperfect pattern element detections can be manually corrected, can substantially accelerate the pace of research surrounding the ecology, evolution, and development of spots and eyespots in butterflies.
