# Correction: Detection and measurement of butterfly eyespot and spot patterns using convolutional neural networks

**DOI:** 10.1371/journal.pone.0294793

**Published:** 2023-11-17

**Authors:** Carolina Cunha, Hemaxi Narotamo, Antónia Monteiro, Margarida Silveira

There are errors in the Funding section. The correct Funding statement is: CC and MS were funded by FCT—Foundation for Science and Technology, Portugal, under project UIDB/50009/2020. https://www.fct.pt/ H.N was funded by Fundação para a Ciência e a Tecnologia (FCT), under Doctoral Grant 2020.04511.BD. https://www.fct.pt/ AM was supported by the National Research Foundation, Singapore, Investigatorship award NRF-NRFI05-2019-0006. https://www.nrf.gov.sg/.
